# TGFβ pathway deregulation and abnormal phospho‐SMAD2/3 staining in hereditary cerebral hemorrhage with amyloidosis‐Dutch type

**DOI:** 10.1111/bpa.12533

**Published:** 2017-06-12

**Authors:** Laure Grand Moursel, Leon P. Munting, Linda M. van der Graaf, Sjoerd G. van Duinen, Marie‐Jose T. H. Goumans, Uwe Ueberham, Remco Natté, Mark A. van Buchem, Willeke M. C. van Roon‐Mom, Louise van der Weerd

**Affiliations:** ^1^ Department of Human Genetics Leiden University Medical Center Leiden the Netherlands; ^2^ Department of Radiology Leiden University Medical Center Leiden the Netherlands; ^3^ Department of Pathology Leiden University Medical Center Leiden the Netherlands; ^4^ Department of Molecular Cell Biology Leiden University Medical Center Leiden the Netherlands; ^5^ Paul Flechsig Institute of Brain Research University of Leipzig Leipzig Germany

**Keywords:** hereditary cerebral hemorrhage with amyloidosis‐Dutch type, cerebral amyloid angiopathy, amyloid β mutation E22Q, phospho‐SMAD2/3 granules, TGFβ, postmortem human brain tissue

## Abstract

Hereditary cerebral hemorrhage with amyloidosis‐Dutch type (HCHWA‐D) is an early onset hereditary form of cerebral amyloid angiopathy (CAA) pathology, caused by the E22Q mutation in the amyloid β (Aβ) peptide. Transforming growth factor β1 (TGFβ1) is a key player in vascular fibrosis and in the formation of angiopathic vessels in transgenic mice. Therefore, we investigated whether the TGFβ pathway is involved in HCHWA‐D pathogenesis in human postmortem brain tissue from frontal and occipital lobes. Components of the TGFβ pathway were analyzed with quantitative RT‐PCR. TGFβ1 and TGFβ Receptor 2 (TGFBR2) gene expression levels were significantly increased in HCHWA‐D in comparison to the controls, in both frontal and occipital lobes. TGFβ‐induced pro‐fibrotic target genes were also upregulated. We further assessed pathway activation by detecting phospho‐SMAD2/3 (pSMAD2/3), a direct TGFβ down‐stream signaling mediator, using immunohistochemistry. We found abnormal pSMAD2/3 granular deposits specifically on HCHWA‐D angiopathic frontal and occipital vessels. We graded pSMAD2/3 accumulation in angiopathic vessels and found a positive correlation with the CAA load independent of the brain area. We also observed pSMAD2/3 granules in a halo surrounding occipital vessels, which was specific for HCHWA‐D. The result of this study indicates an upregulation of TGFβ1 in HCHWA‐D, as was found previously in AD with CAA pathology. We discuss the possible origins and implications of the TGFβ pathway deregulation in the microvasculature in HCHWA‐D. These findings identify the TGFβ pathway as a potential biomarker of disease progression and a possible target of therapeutic intervention in HCHWA‐D.

## INTRODUCTION

Sporadic cerebral amyloid angiopathy (sCAA) is a disease of the elderly due to amyloid β (Aβ) deposition in cerebral leptomeningeal and cortical vessels, and is associated with intracerebral hemorrhages. CAA pathology is a common feature in Alzheimer's disease (AD) and is also a defining pathological feature in hereditary cerebral hemorrhage with amyloidosis‐Dutch type [HCHWA‐D; 22]. HCHWA‐D is caused by a Gln‐to‐Glu substitution at codon 693 of the amyloid precursor protein (APP) gene leading to the formation of the Aβ E22Q peptide, a particularly aggregation‐prone and toxic variant of the Aβ peptide 17. The Dutch mutation results in severe CAA pathology with loss of vascular smooth muscle cells and intracerebral hemorrhage typically between the ages of 40 and 65. Although the correlation between HCHWA‐D carrier status, reduced cerebrovascular function and the clinical phenotype has been studied 40, the exact mechanisms underlying Aβ accumulation in the vessel wall are still largely unknown.

Some earlier studies of HCHWA‐D postmortem brain material have focused on Aβ clearance and deposition in the vasculature by the induction and modification of extracellular matrix (ECM) proteins 15, 39. Transforming growth factor β1 (TGFβ1) has a key role in vascular fibrosis by inducing ECM production in vessels. In postmortem AD brain material, TGFβ1 mRNA levels correlate positively with the extent of CAA pathology 47. Moreover, mouse models of TGFβ1 overexpression in astrocytes or neurons demonstrated that high TGFβ1 levels lead to vascular fibrosis 36, 48. Whether these animal models actually accumulate murine Aβ in the CAA pathology‐like vascular plaques remains controversial, but many *in vitro* studies have shown a role for TGFβ1 in promoting APP and Aβ production by astrocytes 1, 4, 12, 21. Interestingly, TGFβ1 astrocytic overexpression in APP‐overexpressing mice results in a CAA increase with a reduction in parenchymal Aβ plaque load 47.

TGFβ1, 2 and 3 isoforms are expressed in mammals and mediate their cellular effects through the TGFβ type I (TGFBR1) and type II (TGFBR2) receptors. TGFβ is present in an inactive form bound to the ECM and is activated by consecutive cleavage of the latent‐associated‐protein and pro‐domain. Once activated, TGFβ binding to TGFBR2 induces transphosphorylation of the TGFBR1 kinase, which subsequently recruits and phosphorylates the receptor regulated Smad (homolog of Drosophila mothers against decapentaplegic) signal transducing proteins, SMAD2 and/or SMAD3. Phospho‐SMAD2/3 (pSMAD2/3) interacts with the common Smad, SMAD4, and translocate into the nucleus to regulate target gene expression. Genes related to ECM synthesis such as plasminogen activator inhibitor‐1 (PAI‐1), fibronectin (FN1) and collagen (Type I Col1A1 and Type III Col3A1 among others) are typical TGFβ target genes 41, and evidence is mounting that an increase in ECM production is linked to CAA pathology 14, 45.

Accordingly, the aim of this study was to investigate in postmortem material whether the TGFβ pathway is involved in the pathogenesis of HCHWA‐D, based on gene expression levels and histological observations. We specifically investigated in HCHWA‐D if there is a correlation between the deregulation of the TGFβ pathway and the extent of CAA pathology.

## MATERIALS AND METHODS

### Experimental design

HCHWA‐D, healthy controls and sCAA brain material was used in the study as summarized in Table 1. Both frontal and occipital cortex were used in all studies, based on the assumption that the CAA pathology seems more severe in the occipital lobes in HCHWA‐D 23 which therefore is expected to represent a more advanced disease stage compared with the frontal lobe. sCAA individuals were included to investigate whether the Dutch mutation in HCHWA‐D results in a different effect on TGFβ signaling compared to CAA pathology in general. As TGFβ signaling increases with age 9, the control group was age‐matched to the HCHWA‐D patients. sCAA patients were significantly older (see Table 1).

**Table 1:** Demographics of cases and material used in this study. Abbreviations: NDC = non‐demented control; sCAA = sporadic cerebral amyloid angiopathy; HCHWA‐D = hereditary cerebral hemorrhage with amyloidosis‐Dutch type; NBB = Netherlands Brain Bank; LUMC = Leiden University Medical Center; n.a. = not available. HCHWA‐D subjects clinical history in Supporting Information Table S2.

Diagnosis	Source	Code	Age	Gender	PMD*	RTqPCR†	IHC‡	IF§
NDC	LUMC	C1	70	M	6	X	X	X
NDC	LUMC	C9	53	F	15			X
NDC	LUMC	C10	69	M	n.a.			X
NDC	LUMC	C11	51	M	15			X
NDC	LUMC	C12	67	M	29			X
NDC	LUMC	C13	78	F	n.a.			X
NDC	NBB	C2	61	F	10	X	X	X
NDC	NBB	C3	64	F	6	X	X	X
NDC	NBB	C4	56	M	9	X	X	X
NDC	NBB	C5	55	M	8	X	X	X
NDC	NBB	C6	51	M	8	X	X	X
NDC	NBB	C7	57	F	8	X	X	X
NDC	NBB	C14	91	F	4		X	
NDC	NBB	C15	89	F	7		X	
NDC	NBB	C16	83	M	5		X	
NDC	NBB	C17	84	F	6		X	
sCAA	LUMC	S1	68	M	n.a.		X	X
sCAA	LUMC	S2	67	M	n.a.		X	X
sCAA	LUMC	S3	68	M	n.a.		X	
sCAA	LUMC	S4	78	F	n.a.		X	
sCAA	LUMC	S5	73	F	n.a.		X	
sCAA +AD	LUMC	S6	69	F	n.a.		X	
sCAA	LUMC	S7	81	M	n.a.		X	
sCAA	LUMC	S8	74	F	n.a.		X	
sCAA	LUMC	S9	84	M	n.a.		X	
sCAA	LUMC	S10	89	F	n.a.		X	
HCHWA‐D	NBB	H7	71	M	6	X	X	X
HCHWA‐D	NBB	H6	61	M	7	X	X	X
HCHWA‐D	LUMC	H4	55	F	15	X	X	X
HCHWA‐D	LUMC	H5	50	M	19	X	X	X
HCHWA‐D	LUMC	H1	48	M	11	X	X	X
HCHWA‐D	LUMC	H2	57	M	3	X	X	X
HCHWA‐D	LUMC	H3	53	F	6	X	X	X
HCHWA‐D	LUMC	H8	51	M	3		X	X
HCHWA‐D	LUMC	H11	81	F	n.a.		X	
HCHWA‐D	LUMC	H9	67	F	n.a.		X	
HCHWA‐D	LUMC	H10	71	M	n.a.		X	

*Postmortem delay (in hours).

†Quantitative RT‐PCR.

**‡**Immunohistochemistry for pSMAD2/3 quantification.

§Immunohistofluorescence for double staining.

We evaluated TGFβ pathway activation by immunohistochemistry in HCHWA‐D brain tissue, staining for the dually phosphorylated pSMAD2 and/or pSMAD3 indicative of active TGFβ receptor signaling. These studies were performed in 11 HCHWA‐D (age 60.5 years ± 10.7 years), 11 control (age 69.2 years ± 14.9 years) and 10 sCAA (age 74.8 years ± 8.0 years) cases. We also analyzed gene expression levels for several pathway components (RT‐PCR, see Table 2) in a sub‐set of these patients of whom frozen brain was available: 7 HCHWA‐D patients (age 56.4 years ± 7.7 years) and 7 age‐matched controls individuals (age 59.1 years ± 0.1 years). Frozen sCAA material was not available for this measurement.

**Table 2:** Primer list used for qRT‐PCR.

Genebank account number	Name	Primers	Target
NM_000660	TGFB1	5′‐TACCTGAACCCGTGTTGCTC‐3′ 5′‐GTATCGCCAGGAATTGTTGC‐3′	Intron‐spanning (exons 2–3)
NM_003238	TGFB2	5′‐CAATGCCAACTTCTGTGCTG‐3′ 5′‐ATATAAGCTCAGGACCCTGCTG‐3′	Intron‐spanning (exon 6–7)
NM_005901	SMAD2	5′‐GTTTTGAAGCCGTCTATCAGC‐3′ 5′‐TTGTTACCGTCTGCCTTCG‐3′	Intron‐spanning (exon 10–11)
NM_005902	SMAD3	5′‐GAAGATGGAGAAACCAGTGACC‐3′ 5′‐ATTCGGGGATAGGTTTGGAG‐3′	Intron‐spanning (exons 4–5)
NM_005359	SMAD4	5′‐TGGAGCTCATCCTAGTAAATGTG‐3′ 5′‐AGGAAATCCTTTCCGACCAG‐3′	Intron‐spanning (exon 2–3)
NM_005904	SMAD7	5′‐AGGGGGAACGAATTATCTGG‐3′ 5′‐TCGTCTTCTCCTCCCAGTATG‐3′	Intron‐spanning (exon 3–4)
NM_001130916	TGFBR1	5′‐CGTGCTGACATCTATGCAATG‐3′ 5′‐TCAACTGATGGGTCAGAAGG‐3′	Intron‐spanning (exon 7–8)
NM_001024847	TGFBR2	5′‐CTGTGTCGAAAGCATGAAGG‐3′ 5′‐AGTCAACGTCTCACACACCATC‐3′	Intron‐spanning (exon 6–7)
NM_000602	PAI‐1	5′‐CAACTTGCTTGGGAAAGGAG‐3′ 5′‐CGTCTGATTTGTGGAAGAGG‐3′	Intron‐spanning (exons 3–4)
NM_212482	FN1	5′‐GCAGTGGCTGAAGACACAAG 5′‐CCTGCCATTGTAGGTGAATG	Intron‐spanning (exon 7–8)
NM_000088	Col1A1	5′‐ ATGACGTGATCTGTGACGAGAC 5′‐TTCTTGGTCGGTGGGTGAC	Intron‐spanning (exons 2–3)
NM_000090	Col3A1	5′‐GACCTGAAATTCTGCCATCC 5′‐GCATGTTTCCCCAGTTTCC	Intron‐spanning (exons 48–49)
NM_003194	TBP	5′‐CGCCGAATATAATCCCAAGC‐3′ 5′‐GAAAATCAGTGCCGTGGTTC‐3′	Intron‐spanning _ reference gene
NM_000983	RPL22	5′‐TCGCTCACCTCCCTTTCTAA‐3′ 5′‐TCACGGTGATCTTGCTCTTG‐3′	Intron‐spanning _ reference gene
NM_000190	HMBS	5′‐GCAACGGCGGAAGAAAA‐3′ 5′‐CGAGGCTTTCAATGTTGCC‐3′	Intron‐spanning _ reference gene

### Brain tissue

Frontal and occipital human postmortem brain tissue was obtained from the Netherlands Brain Bank and from our hospital (LUMC). Written informed consent was obtained for each donor and all material and data were handled in a coded fashion maintaining patient anonymity according to Dutch national ethical guidelines (Code for Proper Secondary Use of Human Tissue, Dutch Federation of Medical Scientific Societies). The study was approved by the local Ethics Committee.

### Quantitative RT‐PCR

Frozen brain tissue was cut with a sliding microtome (Leica SM2010 R), homogenized with ceramic MagNA lyser beads (Roche) and grinded using a Bullet Blender (Next Advance). RNA was extracted immediately with Aurum Total RNA Mini Kit (Biorad), including removal of remaining genomic DNA by an on‐column DNaseI treatment for 25 minutes. Total RNA was eluted in 60 μL of provided buffer and the RNA content was measured with Nanodrop at 260 nm. All RNA extractions were performed in duplicate and cDNA was synthesized directly after extraction with the Transcriptor First Strand cDNA Synthesis Kit (Roche) using Random Hexamer primers at 65°C. The cDNA was then adjusted and aliquoted at 20 ng/μL. Evaluation of RNA Integrity was performed with on‐chip electrophoresis using an RNA 6000 Nano kit and a Bio‐Analyzer 2100 (Agilent Technologies).

Intron‐spanning primers targeting TGFβ pathway components and target genes (indicated in Table 2) were designed for qPCR using Primer3 Plus software 38. Primer pairs were first spotted into the wells (2.5 pmol of each in 2 µL). The qPCR was performed in a 384 wells plate using 6 ng of cDNA in a PCR master mix (Roche; 1 time PCR buffer with MgCl_2_, 0.2 mM dNTPs, 0.28 U FastStart Taq DNA Polymerase) containing 1 time EvaGreen‐qPCR dye (Biotum) and PCR grade water to a final volume of 8 µL per well. All samples were run in duplicate on the same plate along with three reference genes: Hydroxymethylbilane Synthase (HMBS), Ribosomal Protein L22 (RPL22) and TATA‐Box Binding Protein (TBP). The amplification was performed on a LightCycler 480 (Roche) with an initial denaturation of 10 minutes at 95°C, followed by 45 cycles of 10 s denaturation at 95°C, 30 s annealing at 60°C and 20 s elongation at 72°C. Relative expression of the transcript levels was calculated using LinRegPCR v11.1 32 with the raw fluorescence values as input. Transcript levels were calculated with the Geomean of the biological and technical repeat (four points) normalized with two of the reference genes (HMBS and RLP22). The third reference gene (TBP) was used to check the normalization efficiency and the inter‐plate variance. Changes in relative transcript levels were analyzed in GraphPad Prism version 6.00 using an unpaired two‐sided Student's *t* test. Differences between groups were considered significant when *P* < 0.05.

### Immunohistochemical staining and quantification of pSMAD2/3 in blood vessels

Formalin‐fixed, paraffin‐embedded blocks of brain tissue were cut into serial 5 µm thick sections and mounted on coated glass slides (SuperFrost^®^ Plus, VWR). Deparaffinization in xylene and rehydration through a series of ethanol concentrations were followed by antigen retrieval by cooking for 40 minutes at 0.76 bar steam pressure (Steba DD 1 ECO) in an acidic pH 6 solution (H‐3300, Vector labs). Sections were then blocked for endogenous peroxidase with 3% H_2_0_2_ in dH_2_0 for 10 minutes and for unspecific epitopes binding with blocking buffer [1% BSA suspension in washing buffer (0.1% Tween 20 in Phosphate Buffer Saline pH 7.4)] for 1 h at room temperature. After, the sections were incubated with rabbit anti‐pSMAD2/3 antibody (#3101S, Cell Signaling, 1:500 dilution) overnight at 4°C in the blocking buffer. Incubation with secondary anti‐Rabbit HRP was followed by a DAB reaction kit (SK‐4100, Vector lab) and mounting with Entellan^®^ New (107961, Merck). Sections were scanned (Philips Ultra Fast Scanner 1.6 RA) for grading. Collagen IV and laminin staining followed identical procedure (details in Supporting Information Table S1).

The grading of pSMAD2/3 staining was reproduced on scanned sections by two independent researchers (LvdG; JMdJ) blinded to the clinical diagnosis of each case. Six fields throughout gray matter areas of the slides were randomly selected at 100× magnification (2.016 mm^2^ per field view). Per area, radially crosscut parenchymal angiopathic arterioles were counted. CAA load is defined here as the average number of angiopathic arterioles, identified by a typical thickened vessel wall, per mm^2^. Presence of Aβ in angiopathic arterioles was checked on a consecutive slide (data not shown, not used for the grading). pSMAD2/3 deposits in the tunica media of angiopathic arterioles was determined for each vessel at 400× magnification. Difference in CAA load between frontal and occipital cortex was assessed with GraphPad Prism version 6.00 using a paired two‐sided Student's *t* test. Differences between groups were considered significant when *P* < 0.05.

### Immunohistofluorescent double staining and pSMAD2/3 specificity

Formalin‐fixed, paraffin‐embedded frontal and occipital cortex 5 µm sections were used from patient material as specified in Table 1. Deparaffinization, antigen retrieval and blocking steps were identical to the immunohistochemical staining. Rabbit anti‐pSMAD2/3 antibody (#3101S, Cell Signaling; 1:500 dilution) was incubated overnight at 4°C with mouse antibodies (references and dilutions in Supporting Information Table S1). The antibodies were visualized with anti‐rabbit Alexa Fluor^®^ 488 and anti‐mouse Alexa Fluor^®^ 594, respectively (1 h at RT). Alternatively, Tyramide Signal Amplification (TSA^®^ biotin detection kit, NEL700A001KT, Perkin Elmer) followed by streptavidin, Alexa Fluor^®^ 488 conjugate was used when specified in the text (Supporting Information Table S1). Nuclei were stained with DAPI (1 μg/mL) during the secondary antibody incubation step. After each incubation, the slides were extensively washed in washing buffer. Sections were mounted in Pro Long Diamond (Life technologies). Images of the fluorescent staining were acquired using a confocal laser‐scanning microscope (Leica SP8, Leica Microsystems). The specificity of pSMAD2/3 staining was assessed either with calf intestine alkaline phosphatase treatment removing the phosphor‐epitopes [adapted from Ref. ( 37), details in Supporting Information Figure S1] and by using different phospho‐antibodies for the same epitope (Supporting Information Figure S1 and Table S1). No cross‐reactivity were observed with anti‐pSMAD1/5/9 (data not shown). A granular perivascular pSMAD2/3 staining was also detected by Dr. Ueberham.

## RESULTS

### TGFβ1 and TGFBR2 are upregulated in HCHWA‐D

**Figure 1:**
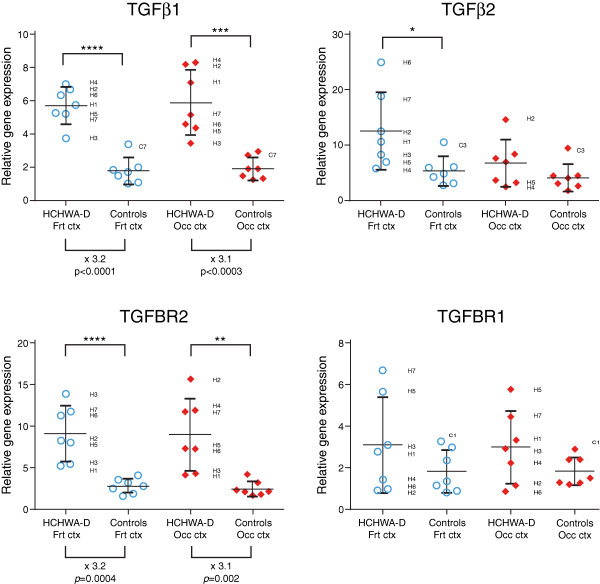
Significant upregulation of TGFβ1 and TGFBR2 gene levels (left panel; x indicates the time fold change) in HCHWA‐D frontal (Frt) and occipital (Occ) cortex compared to age‐related controls. Transcript expression levels in postmortem brain cortex were normalized with two reference genes and represented in a dot plot with mean ± SD of seven samples; *P < 0.05, **P < 0.01, ***P < 0.001 and ****P < 0.0001 as determined by a two‐tailed unpaired Student's *t* test.

From literature we know that the SMAD‐dependent signaling TGFβ pathway is activated upon binding of TGFβ1 or TGFβ2 to TGFBR2, followed by phosphorylation of SMAD2 and 3 by TGFBR1. To assess TGFβ pathway implication we measured gene expression levels of TGFβ1, TGFβ2, TGFBR1, TGFBR2, SMAD2, SMAD3, SMAD4 and SMAD7 by qRT‐PCR. The relative gene expression levels of TGFβ1 and TGFBR2 were significantly higher in the frontal and occipital lobes of HCHWA‐D patients compared to age‐related controls (Figure 1). TGFBR1 followed a similar trend, but did not reach statistical significance. TGFβ2 levels were significantly higher in the frontal lobe of HCHWA‐D samples, especially in the two eldest patients from the gene expression study (H6 and H7); (Figure 1). Interestingly, these two samples also present the highest level of the inhibitory Smad, SMAD7, suggesting an enhanced TGFβ pathway activation compared to the other HCHWA‐D patients (Supporting Information Figure S2). Other signaling effectors of the canonical SMAD pathway (SMAD2, SMAD3, SMAD4 and SMAD7) were further not significantly different (Supporting Information Figure S2).

**Figure 2:**
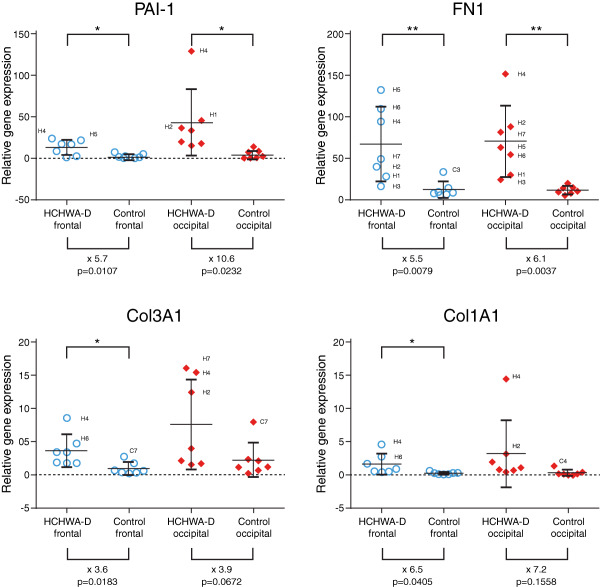
Significant upregulation of PAI‐1 and FN1 gene levels (upper panel; x indicates the time fold change) in HCHWA‐D frontal (Frt) and occipital (Occ) cortex compared to age‐related controls. Col3A1 and Col1A1 increase reach statistical significance in frontal cortex only. Transcript expression levels in postmortem brain cortex were normalized with two reference genes and represented in a dot plot with mean ± SD of seven samples; *P < 0.05, **P < 0.01, ***P < 0.001 and ****P < 0.0001 as determined by a two‐tailed unpaired Student's *t* test.

Plasminogen activator inhibitor‐1 (PAI‐1), fibronectin1 (FN1), Col1A1 and Col3A1 are known SMAD‐dependent downstream targets of TGFβ. PAI‐1 and FN1 gene expression levels were both significantly higher in frontal and occipital cortex of HCHWA‐D patients. Col3A1 and Col1A1 were upregulated in both brain area, but reach statistical significance in the frontal cortex only (Figure 2).

### pSMAD2/3 is accumulating in HCHWA‐D, but not in sCAA

To evaluate TGFβ pathway activation by immunohistochemistry in HCHWA‐D brain tissue, we stained for the TGFβ1 down‐stream signaling effector dually phosphorylated pSMAD2/3. Frontal and occipital cortex of controls, sCAA and HCHWA‐D cases were assessed for pSMAD2/3 staining. In all the observed cases, pSMAD2/3 labeling was predominantly located in nuclei of neurons, with little or no cytoplasmic staining detected in both brain area.

**Figure 3:**
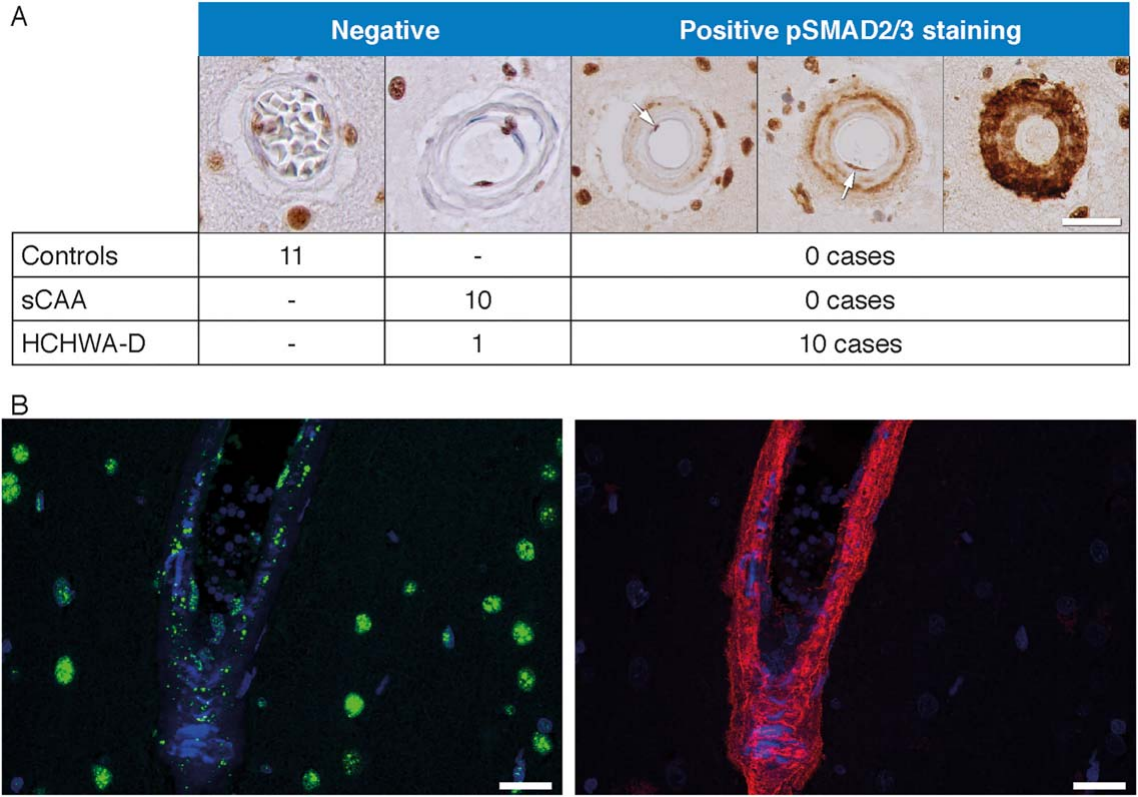
pSMAD2/3 granular deposits in angiopathic vessel walls. **A.** Examples of immunohistochemical pSMAD2/3 staining graded as negative and positive. pSMAD2/3 deposits were found in 10 out of 11 HCHWA‐D cases (at least one positive vessel per slide), none were found in controls or sCAA cases. Staining of endothelial cell nuclei are not counted (see the “negative” example). (arrows illustrate nuclei of endothelial cells in luminal position). **B.** Immunohistofluorescent double staining with pSMAD2/3 TSA (green), Aβ 6E10 (red) and nuclei (blue). pSMAD2/3 deposits in parenchymal angiopathic arterioles, with Aβ covering the entire vessel circumference. HCHWA‐D H2 patient‐occipital cortex, merged confocal stack. Scale bar (**A**, **B**) 25 μm.

Both in frontal and occipital cortex, pSMAD2/3 staining in amyloid‐laden vessels were found uniquely in HCHWA‐D cases. The staining pattern showed distinct granules in the tunica media as well as diffuse staining covering the entire vessel wall. Examples of these granular deposits are given in Figure 3A. pSMAD2/3‐positive and negative vessels were not morphologically different. Notably, pSMAD2/3 deposits were mainly found in parenchymal arterioles with Aβ covering the entire vessel circumference (double staining with anti‐Aβ antibodies, Figure 3B), corresponding to an advanced CAA grade [grade 2 or 3 based on the Aβ content as defined by Greenberg and Vonsattel 13]. Nevertheless, these granular deposits in the tunica media were not colocalized with the smooth muscle actin (SMA) staining, and we could observe in some angiopathic vessels an accumulation of pSMAD2/3 granules in vacuoles believed to be the remains of vascular smooth muscle cells 25 (Supporting Information Figure S3).

### pSMAD2/3 accumulation is correlated with CAA load, but less with age

**Figure 4:**
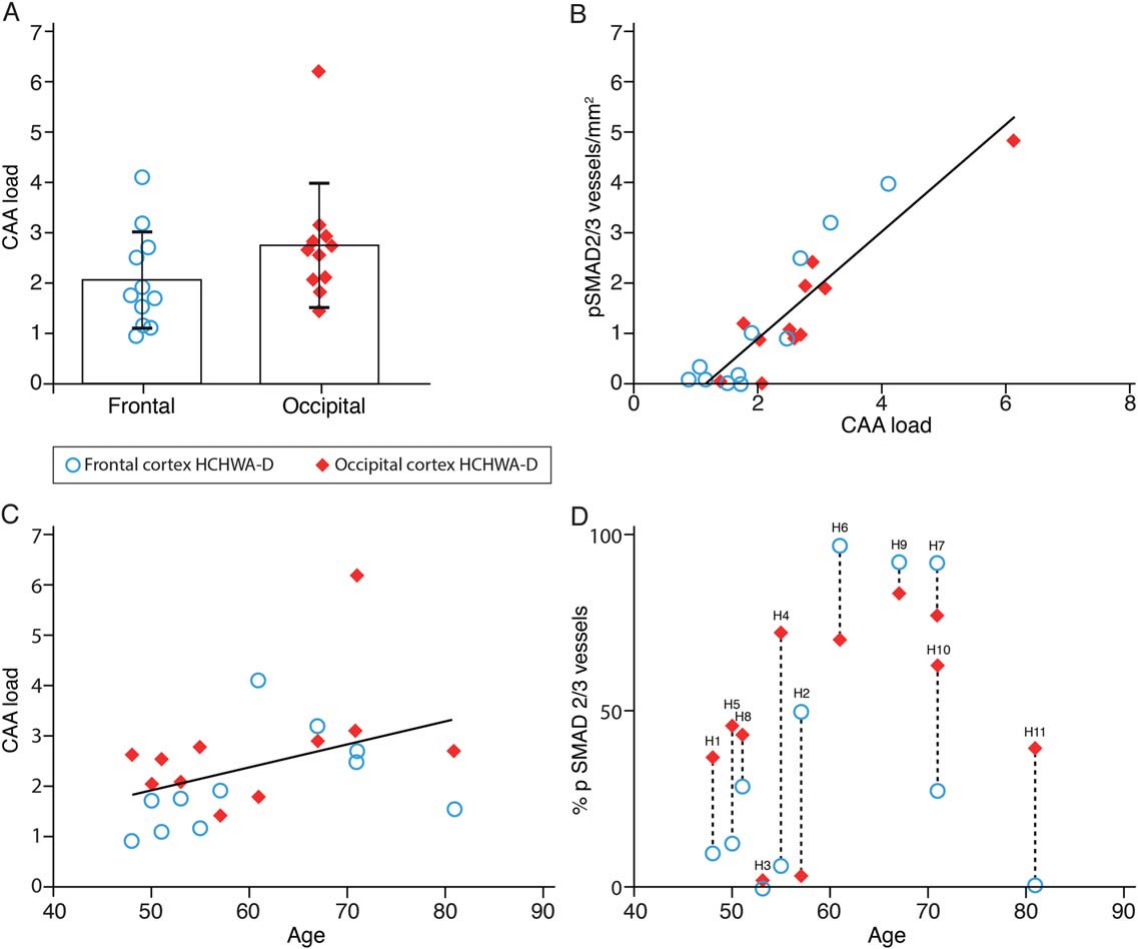
Quantification of CAA load and relationship with pSMAD2/3 accumulation in HCHWA‐D. **A.** CAA load was higher in occipital cortex but did not reach statistical difference (two‐tailed paired Student's *t* test; the graph represents the mean ± SD of angiopathic vessels per mm^2^). **B.** Angiopathic pSMAD2/3 vessel wall deposits were correlated positively with CAA load (both expressed as number of vessel per mm^2^; *r* ^2^ = 0.8220, *P* < 0.0001). **C.** CAA load was weakly correlated with age (*r* ^2^ = 0.1702; not statistically significant). **D.** Proportion of angiopathic vessels in HCHWA‐D subjects with pSMAD2/3 vessel wall deposits (dashed lines link brain parts of the same subject; H numbers refer to Table 1).

Entire tissue sections were graded to assess pSMAD2/3 staining in HCHWA‐D samples. For the same sections, the CAA load was measured as the number of angiopathic arterioles in a defined area. We found no significant differences in CAA load between frontal and occipital cortex (Figure 4A). However, a significant positive correlation between pSMAD2/3‐positive angiopathic vessels and CAA load was found independently of the brain area studied (Figure 4B). This confirmed that pSMAD2/3 deposits are only present once the CAA pathology becomes more severe. In areas with a high CAA load, most angiopathic vessels are pSMAD2/3‐positive. Although there is a strong correlation between pSMAD2/3 and CAA pathology severity, CAA load in itself is only moderately dependent on the age of the patients (Figure 3C,D). In particular, the 81‐years‐old patient, who reached an unusual age for HCHWA‐D, was much less affected than expected.

### Perivascular pSMAD2/3 granules are present in occipital cortex of HCHWA‐D

**Figure 5:**
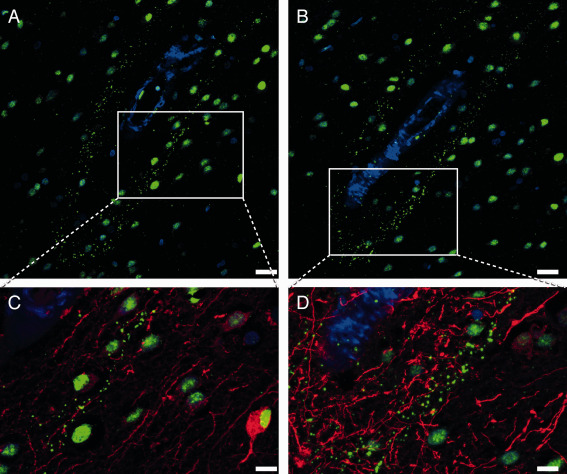
Perivascular pSMAD2/3 granules showed a linear alignment in a vessel with a longitudinal cut in HCHWA‐D occipital cortex. Immunohistofluorescent double staining with neuronal (MAP‐2) and astrocytic (GFAP) cytoskeleton markers. **A‐B.** pSMAD2/3 TSA (green) and nuclei (blue) channels. **C‐D.** Detail including MAP‐2 (red; **C**) or GFAP (red; **D**). Presence of perivascular pSMAD2/3 granules along the neuronal MAP‐2 dendrites (**C**), and never colocalizing with GFAP processes (**D**). Consecutive sections of HCHWA‐D H1 patient‐occipital cortex, merged confocal stack. Scale bar (**A‐B**) 25 μm; (**C‐D**) 10 μm.

Apart from granules localized on angiopathic vessels in HCHWA‐D, pSMAD2/3 granules were also found in the parenchyma. These were observed as perivascular parenchymal rings (see Figure 5A,B) in the occipital cortex in about 50% of the cases (Supporting Information Table S2), but never in the frontal cortex of HCHWA‐D brains or in sCAA and control cases. Perivascular rings were found both around angiopathic and non‐angiopathic vessels, as well as around capillaries, and were found in clusters with a predilection for the first and last cortical layer of the gray matter. SMAD4 binding to pSMAD2/3 is a prerequisite for SMAD2/3 to enter the nucleus and initiate gene transcription. However, in our samples, SMAD4 was neither co‐localized with granular vascular, nor with perivascular halo of pSMAD2/3 staining (Supporting Information Figure S4); questioning the participation of the granules in the active signaling.

### Perivascular pSMAD2/3 granules do not co‐localize with studied cell types or neuropathological features

**Figure 6:**
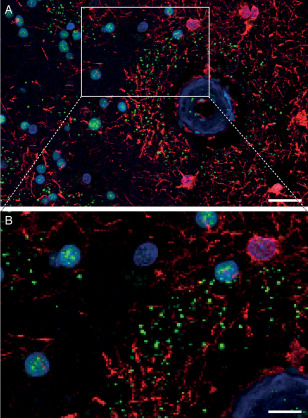
Perivascular ring of pSMAD2/3 granules limited to the area of GFAP‐positive astrocytes. Immunohistofluorescent double staining with pSMAD2/3 (green), GFAP (red) and nuclei (blue). **B** detail of **A**. HCHWA‐D H1 patient‐occipital cortex, merged confocal stack. Scale bar: **A.** 25 μm; **B.** 10 μm.

Since perivascular pSMAD2/3 granules were arranged in a linear alignment, reminiscent of cytoskeletal filaments (Figure 5), we investigated whether the granules co‐localized with a particular cell type using neuronal and astrocytic cytoskeleton markers. MAP‐2 staining revealed a presence of the granules along perivascular dendrites (Figure 5C) without co‐localization within the neuronal processes. pSMAD2/3 granules never aligned with GFAP processes (Figure 5D) but were topographically restricted to GFAP‐positive perivascular areas, as depicted in Figure 6. Further, parenchymal cytoplasmic pSMAD2/3 granules were incidentally identified in neurons in some HCHWA‐D individuals (Supporting Information Figure S5).

**Figure 7:**
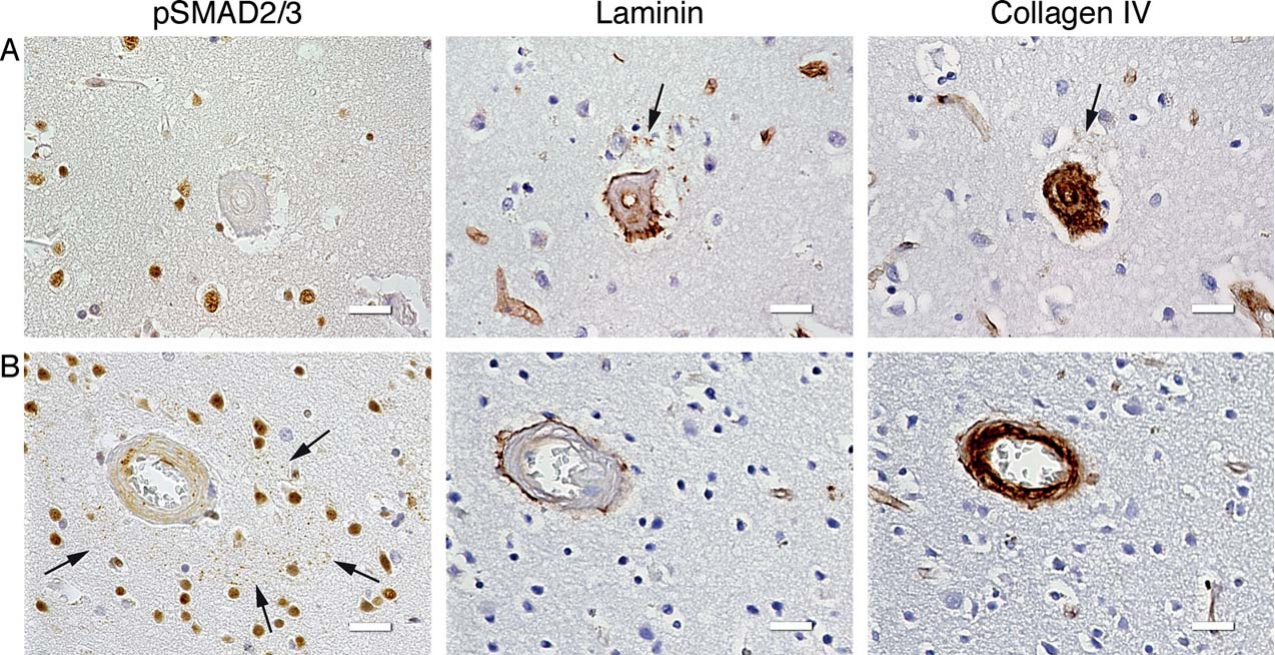
ECM coarse deposits did not spatially correspond to the pSMAD2/3 granules. Immunohistochemical staining of consecutive slides with pSMAD2/3, laminin and collagen IV antibodies. **A.** Laminin coarse deposit (and weak collagen IV colocalization; arrows) without presence of pSMAD2/3 granules; HCHWA‐D H5 patient‐occipital cortex. **B.** pSMAD2/3 perivascular ring of granules (arrows) without perivascular ECM deposits; HCHWA‐D H1 patient‐occipital cortex. Scale bar 25 μm.

Previous HCHWA‐D studies reported perivascular ubiquitinated and phosphorylated neurites associated with preamyloid and amyloid deposits around angiopathic vessels 24, 33. Therefore, we examined in HCHWA‐D occipital samples whether the pSMAD2/3 perivascular granules were co‐localized with any of these previously reported deposits. No co‐localization of pSMAD2/3 granules was found in the perivascular rings with Aβ, hyperphosphorylated Tau (pTau, AT‐8 antibody) and ubiquitin. Likewise, we stained for perivascular coarse deposits of ECM as described previously for HCHWA‐D using collagen IV and laminin antibodies 39. Although we found these coarse deposits, they did not co‐localize with pSMAD2/3 granules (Figure 7A,B). Furthermore, an extracellular pSMAD2/3 deposition with amyloid deposits and neuritic plaques was described in AD 6, 37. Even though a similar co‐localization with diffuse parenchymal Aβ plaques in HCHWA‐D was occasionally detected, these extracellular pSMAD2/3 deposits had a fibrous‐like and diffuse staining that is different from the bright round‐shaped dots composing the pSMAD2/3 granular rings (Supporting Information Figure S6).

## DISCUSSION

Our findings suggest that TGFβ is implicated in the pathogenesis of HCHWA‐D. We found an upregulation in gene expression of several components of the TGFβ pathway and its direct downstream signaling targets as well as a strong correlation of pSMAD2/3 deposits with CAA load.

Based on previous studies in HCHWA‐D describing an increased CAA pathology in the occipital cortex compared to the frontal cortex 23, we hypothesized that the CAA load and the associated vascular pathology in the occipital lobe would represent a more advanced disease stage. However, in our HCHWA‐D samples, individual perforating arterioles presented a consistent moderate to severe CAA grade (grade 2 to 3), irrespective of the brain area studied. Furthermore, the CAA load, based on the number of angiopathic vessels per mm^2^, was not significantly different in the two lobes (Figure 4A). A possible explanation for this finding is that the higher CAA load in the occipital cortex compared to the frontal cortex in previous studies is influenced by the presence of angiopathic capillaries 25. Capillary CAA is typically a feature of aged patients, while our cohort was relatively young. Additionally, the high occipital CAA load in the occipital cortex was in most cases confined to the end of the occipital horn 28, whereas our samples were obtained from diverse occipital area.

Although we did not find a difference in CAA severity between occipital and frontal lobes, we found a high correlation between the CAA load and the presence of pSMAD2/3 granules. Strikingly, these granules were only found in HCHWA‐D patients, but not in sCAA cases, even in similarly affected vessels. The association of CAA pathology with pSMAD2/3, which is a direct TGFβ down‐stream signaling effector, suggests an involvement of TGFβ on the vessel wall pathology. TGFβ is thought to be a key mediator of vascular remodeling 31 that is found in sporadic small‐vessel diseases 34. TGFβ deregulation in the vessel wall is a central mechanism common to several hereditary brain microvasculopathies, like cerebral autosomal dominant arteriopathy with subcortical infarcts and leukoencephalopathy [CADASIL; 18] or cerebral autosomal recessive arteriopathy with subcortical infarcts and leukoencephalopathy [CARASIL, 3, 49]. TGF‐β signaling was recently proposed as a common denominator of several forms of cerebral small‐vessel diseases 26. Common microvasculopathies include microaneurysms, fibrinoid necrosis, obliterative intimal changes, and hyaline thickening. Interestingly, these secondary structural microvasculopathies, are found more frequently in HCHWA‐D in comparison to sCAA 42 and are correlated to CAA load 28. This aggravated remodeling may be due to a fast progression of the disease in HCHWA‐D or to a direct effect of Dutch‐type Aβ binding to TGFBR2, directly activating the signaling pathway 16. Accumulation of pSMAD2/3 granules in the vessel wall in HCHWA‐D supports the hypothesis that TGFβ deregulation contributes to secondary microvascular remodeling.

TGFβ1 was upregulated in our HCHWA‐D postmortem brain tissue, similar to a previous study where a correlation was found in the cerebrovascular pathology of AD 46. Although part of the observed result is possibly influenced by differences in the cellular composition of HCHWA‐D cortex, normalization per cell type cannot be achieved, nor is commonly done in RT‐qPCR. We corrected for the total amount of transcript per sample with stably expressed references gene. Another potential confounder in our study is that all patients suffered one or more hemorrhagic strokes before death, which influences TGFβ1 expression. It is known that the expression of TGFβ1 increases rapidly after brain injury to restrict brain damage and as part of the healing process 9, 11. Still, we found consistent upregulation in all patients in our cohort, despite very different survival times after hemorrhage (from days to several years), variable hemorrhage sites and even unrelated causes of death. This suggests that the TGFβ upregulation we found cannot solely be explained by an acute response after stroke, but is likely linked to the CAA pathology itself, as evidenced by the histological spatial correlation described above. We also found TGFBR2, the ligand‐binding receptor, upregulated in HCHWA‐D occipital and frontal cortex. Previous studies have also found high levels of TGFBR2 in a mouse model of traumatic cerebral injury and stroke in the chronic phase 10, 30. These findings indicate that TGFBR2 upregulation is not an acute response. Last, the upregulation of TGFβ‐induced pro‐fibrotic target genes such as PAI‐1, FN1, Col1A1 and Col3A1 41 indicate that the TGFβ signaling pathway is likely activated.

TGFβ upregulation is a double‐edged sword with both protective and deleterious consequences. The vascular remodeling and fibrosis induced by TGFβ might have a protective effect in CAA pathology, due to the cross‐linked increased ECM and basement membrane which might prevent the weakening of the vessel wall 8, 50. Despite this protective effect in terms of stroke survival and prevention of hemorrhage, persistent TGFβ upregulation is also thought to have a major downside. The resultant ECM synthesis which modifies the composition of basement membrane impairs perivascular drainage, thereby triggering further amyloid deposition and aggravating the CAA 5, 43. This was demonstrated in mouse models of both inducible neuronal‐ and astrocytic‐TGFβ overexpression, where perivascular astrogliosis is preceding and promoting the vascular angiopathy 36, 48.

In our study, we found a perivascular ring of pSMAD2/3 granules around vessels in the occipital lobe of HCHWA‐D samples. Comparable pSMAD2/3 granules were found in other neurodegenerative disorders, such as AD, Pick's syndrome, progressive supranuclear palsy and corticobasal degeneration 2, 6, 7. Typically, in AD the granules were found intracellularly and associated with neuronal aggregates of pTau or granulovacuolar inclusion of ubiquitin 2, 20, 27, 37. This aberrant cytoplasmic dislocation of pSMAD2/3 could impair the normal TGFβ signaling pathway by sequestration of this transcription factor 2, 6, 27, 37. In our study, we did not find pTau, ubiquitin or Aβ colocalization with the perivascular ring of pSMAD2/3 granules, which is not surprising considering the general lack of neuronal degeneration and pTau involvement in HCHWA‐D. Nevertheless, the granular sequestration of pSMAD2/3 may point to a similar deregulation of the TGFβ pathway.

In the current study, we often found that the perivascular granules were positioned following a linear pattern, reminiscent of astrocytic processes. In a recent study, a decrease in cerebrovascular reactivity in the occipital cortex of HCHWA‐D was described as an early biomarker of the disease 40 and astrocytes are key mediators in this process 19. In transgenic mice with cerebral angiopathy due to overexpression of TGFβ or APP, the cerebrovascular reactivity was impaired, due to neurovascular decoupling 29, 35. This decoupling is the result of retraction of astrocyte end feet from the vessel wall. Similar underlying mechanisms likely occur in HCHWA‐D patients. Such perivascular astrocytic remodeling has been linked in AD mouse models with CAA pathology severity and astrocytic phenotypic switch, defined by a loss of GFAP‐positivity 44. The perivascular granules could be the remnants of astrocytic cytoskeletal remodeling.

In summary, our results indicate a possible contribution of TGFβ to the amyloid angiopathy and the resulting vascular remodeling seen in HCHWA‐D. Future studies into the early involvement of TGFβ in the amyloid angiopathy pathogenesis should be determined in ongoing longitudinal studies in HCHWA‐D.

## COMPLIANCE WITH ETHICAL STANDARDS

**Conflicts of Interest**: The authors declare they have no conflicts of interest.

**Ethical approval**: All procedures performed in this study involving human participants were in accordance with the ethical standards of the institutional and/or national research committee and with the 1964 Helsinki declaration and its later amendments or comparable ethical standards.

**Informed consent**: Informed consent was obtained from all individual participants included in the study.

## Supporting information

Additional Supporting Information may be found in the online version of this article at the publisher's web‐site:

**Figure S1.** Specificity of the pSMAD2/3 staining. (A‐B) Phosphatase treatment hampered the immunohistochemical pSMAD2/3 staining. (A) Phosphatase treated*. (B) Non‐treated consecutive slide. (C‐D) Perivascular granules are detected with two other different antibodies (detailed in Supplementary Table 1). (C) 11769‐R Santa Cruz, immunohistofluorescent staining with pSMAD2/3 TSA (*green*) and nuclei (*blue*). (D) #3108 Cell signaling, immunohistochemical pSMAD2/3 staining. HCHWA‐D H1 occipital cortex. *Scale bar* 50 μm.
**Figure S2.** No significant upregulation of SMAD transcription factors gene levels in HCHWA‐D frontal and occipital cortex compared to age‐related controls. Transcript expression levels in postmortem brain cortex were normalized with two reference genes and represented in a dot plot with mean ± SD of seven samples.
**Figure S3.** pSMAD2/3 granular deposits in angiopathic vessel walls are posterior to vascular smooth muscle cells (VSMCs) disappearance. (**A**) Immunohistochemical pSMAD2/3 staining (pSMAD2/3 TSA (*green*), SMA (*red*) and nuclei (*blue*). (**B and C**) detail of (**A**). pSMAD2/3 granules are present on the vessel wall in the absence of smooth muscle actin (SMA) staining (*arrows*) (**B**); but were not colocalizing with SMA staining in remnant vascular smooth muscle cells (VSMCs) (*arrows*) (**C**). HCHWA‐D H1 patient‐occipital cortex, epifluorescence microscope, Leica DM5500. *Scale bar* (**A**) 50 nm (**B**;**C**) 10 nm (**D**) Immunohistofluorescent double staining with pSMAD2/3 (*green*), GFAP (*red*) and nuclei (*blue*). (**E**) detail of (**D**). pSMAD2/3 granular deposits are accumulating in the tunica media, here in vacuoles, believed to be the remains of the VSMCs (*arrows*) (**E**). HCHWA‐D H2 patient‐occipital cortex, merged confocal stack. *Scale bar* (**D**) 25 nm (**E**) 10 nm.
**Figure S4.** SMAD4 did not colocalize with pSMAD2/3 granules neither at the perivascular ring (*star*), nor on the vessel wall (*arrow*). (A) immunohistochemical pSMAD2/3 staining (TSA enhancement with DAB procedure, *brown*). (B) immunohistochemical SMAD4 staining (*brown)*. HCHWA‐D H1 occipital cortex. *Scale bar* 25 μm.
**Figure S5.** In HCHWA‐D, a rare example of parenchymal cytoplasmic granulo‐vesicular pSMAD2/3 (*arrow*) in a neuron (shape‐based identification) associated with reduced nuclear signaling, as described in AD 2, 6, 38. Immunohistofluorescent staining with pSMAD2/3 TSA (*green*) and nuclei (*blue*) channels. HCHWA‐D H4 patient‐occipital cortex. *Scale bar* 10 μm.
**Figure S6.** Extra‐cellular pSMAD2/3 deposits co‐localized with diffuse parenchymal Aβ plaque (*star*), like in AD 6, 38, but are different from the bright round‐shaped dots composing the perivascular granular ring (*arrow*). Immunohistofluorescent double staining with pSMAD2/3 TSA (*green*), Aβ 6E10 (*red*) and nuclei (*blue*). (A) Merged confocal stack, (B) detail of (A). HCHWA‐D H1 patient‐occipital cortex. *Scale bar* (A) 25 μm; (B) 10 μm.

**Table S1.** List of antibodies used in this study.
**Table S2.** Occurrence of pSMAD2/3 deposits in occipital cortex of HCHWA‐D subjects and clinical details.
