# Supplementary material for: TGFβ pathway deregulation and abnormal phospho‐SMAD2/3 staining in hereditary cerebral hemorrhage with amyloidosis‐Dutch type
Source: Brain Pathol. 2017 Jun 12;28(4):495–506. doi: 10.1111/bpa.12533 (PMC8028662; doi:10.1111/bpa.12533)
Supplement: Supplementary file 1 — Figure S1. Specificity of the pSMAD2/3 staining. (A‐B) Phosphatase treatment hampered the immunohistochemical pSMAD2/3 staining. (A) Phosphatase treated*. (B) Non‐treated consecutive slide. (C‐D) Perivascular granules are detected with two other different antibodies (detailed in Supplementary Table 1). (C) 11769‐R Santa Cruz, immunohistofluorescent staining with pSMAD2/3 TSA (green) and nuclei (blue). (D) #3108 Cell signaling, immunohistochemical pSMAD2/3 staining. HCHWA‐D H1 occipital cortex. Scale bar 50 μm. Figure S2. No significant upregulation of SMAD transcription factors gene levels in HCHWA‐D frontal and occipital cortex compared to age‐related controls. Transcript expression levels in postmortem brain cortex were normalized with two reference genes and represented in a dot plot with mean ± SD of seven samples. Figure S3. pSMAD2/3 granular deposits in angiopathic vessel walls are posterior to vascular smooth muscle cells (VSMCs) disappearance. (A) Immunohistochemical pSMAD2/3 staining (pSMAD2/3 TSA (green), SMA (red) and nuclei (blue). (B and C) detail of (A). pSMAD2/3 granules are present on the vessel wall in the absence of smooth muscle actin (SMA) staining (arrows) (B); but were not colocalizing with SMA staining in remnant vascular smooth muscle cells (VSMCs) (arrows) (C). HCHWA‐D H1 patient‐occipital cortex, epifluorescence microscope, Leica DM5500. Scale bar (A) 50 nm (B;C) 10 nm (D) Immunohistofluorescent double staining with pSMAD2/3 (green), GFAP (red) and nuclei (blue). (E) detail of (D). pSMAD2/3 granular deposits are accumulating in the tunica media, here in vacuoles, believed to be the remains of the VSMCs (arrows) (E). HCHWA‐D H2 patient‐occipital cortex, merged confocal stack. Scale bar (D) 25 nm (E) 10 nm. Figure S4. SMAD4 did not colocalize with pSMAD2/3 granules neither at the perivascular ring (star), nor on the vessel wall (arrow). (A) immunohistochemical pSMAD2/3 staining (TSA enhancement with DAB procedure, brown). (B) immunohistochemi [file BPA-28-495-s002.pdf]

Supplementary material

Article title: TGFβ pathway deregulation and abnormal phospho-SMAD2/3 staining in hereditary cerebral hemorrhage with amyloidosis-Dutch type

Journal: Brain Pathology (2017)

Authors and affiliations: Laure Grand Moursel*[1,2], Leon P. Munting[1,2], Linda M. van der Graaf[1,2], Sjoerd G. van Duinen[3], Marie-Jose T.H. Goumans[4], Uwe Ueberham[5], Remco Natté[3], Mark A. van Buchem[2], Willeke M.C. van Roon-Mom[1], Louise van der Weerd[1,2]

[1]Department of Human Genetics, Leiden University Medical Center
[2]Department of Radiology, Leiden University Medical Center
[3]Department of Pathology, Leiden University Medical Center
[4]Department of Molecular Cell Biology, Leiden University Medical Center
[5]Paul Flechsig Institute of Brain Research, University of Leipzig

***Corresponding author**
Leiden University Medical Center, Einthovenweg 20, 2333 ZC Leiden, The Netherlands
Phone: +31-71-526 9603 E-mail: L.Grand_Moursel@lumc.nl

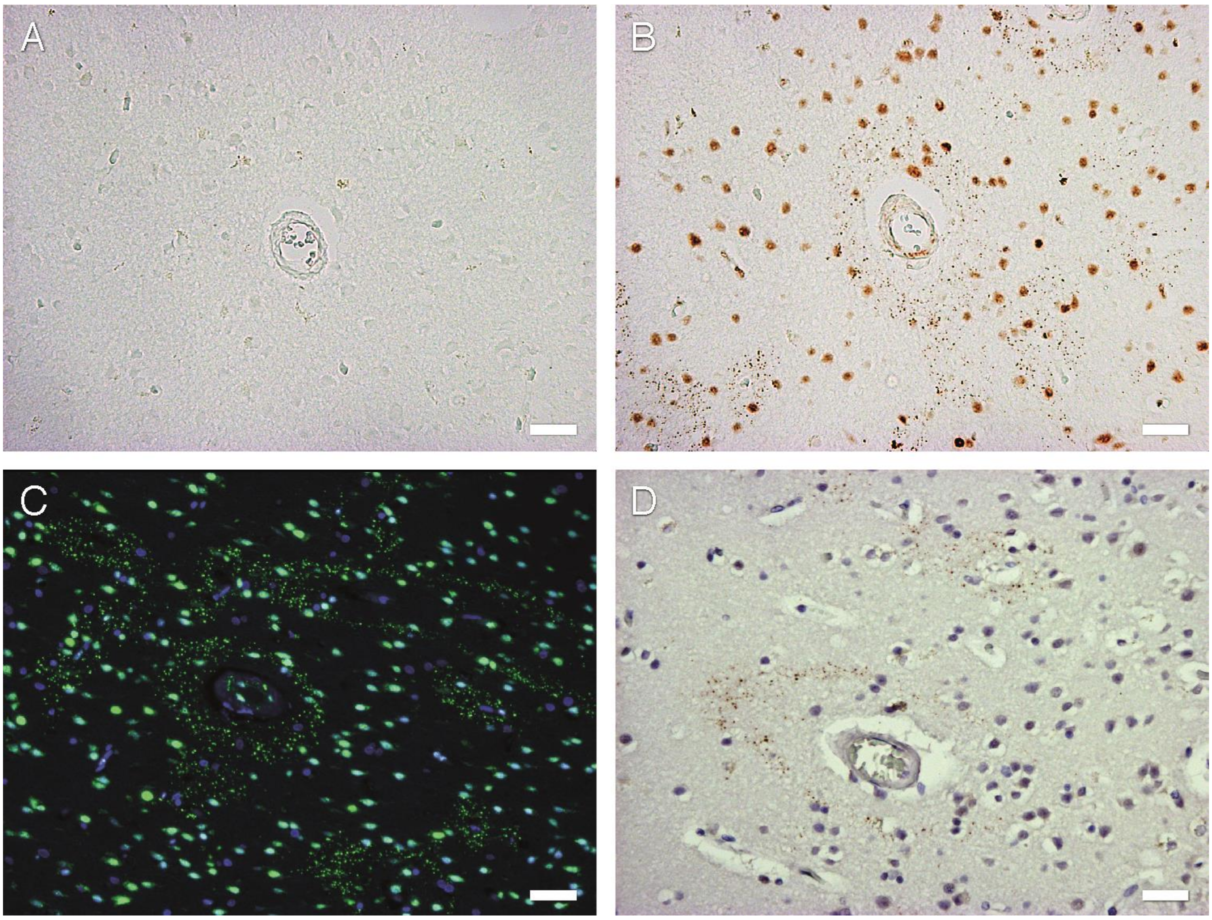

Supplementary Fig. 1 Specificity of the pSMAD2/3 staining. (A-B) Phosphatase treatment hampered the immunohistochemical pSMAD2/3 staining. (A) Phosphatase treated*. (B) Non treated consecutive slide. (C-D) Perivascular granules are detected with two other different antibodies (detailed in Supplementary Table 1). (C) 11769-R Santa Cruz, immunohistofluorescent staining with pSMAD2/3 TSA (*green*) and nuclei (*blue*). (D) #3108 Cell signalling, immunohistochemical pSMAD2/3 staining. HCHWA-D H1 occipital cortex. *Scale bar* 50µm

*Method (adapted from [38]): Phosphatase treatment was applied in between the blocking step and primary antibody incubation. Phosphatase treated slide was incubated with 100U Calf Intestine Alkaline Phosphatase (CIAP; 79390 SIGMA) in 50mM Tris pH10 solution for 2 hours at 37°C. Non treated slide was simultaneously incubated with 1x PhosStop (EASYpack Roche) in 50mM Tris pH10 solution. The slides were then washed twice in PBS with 0,1% Tween 20 before continuation with the immunohistochemistry protocol

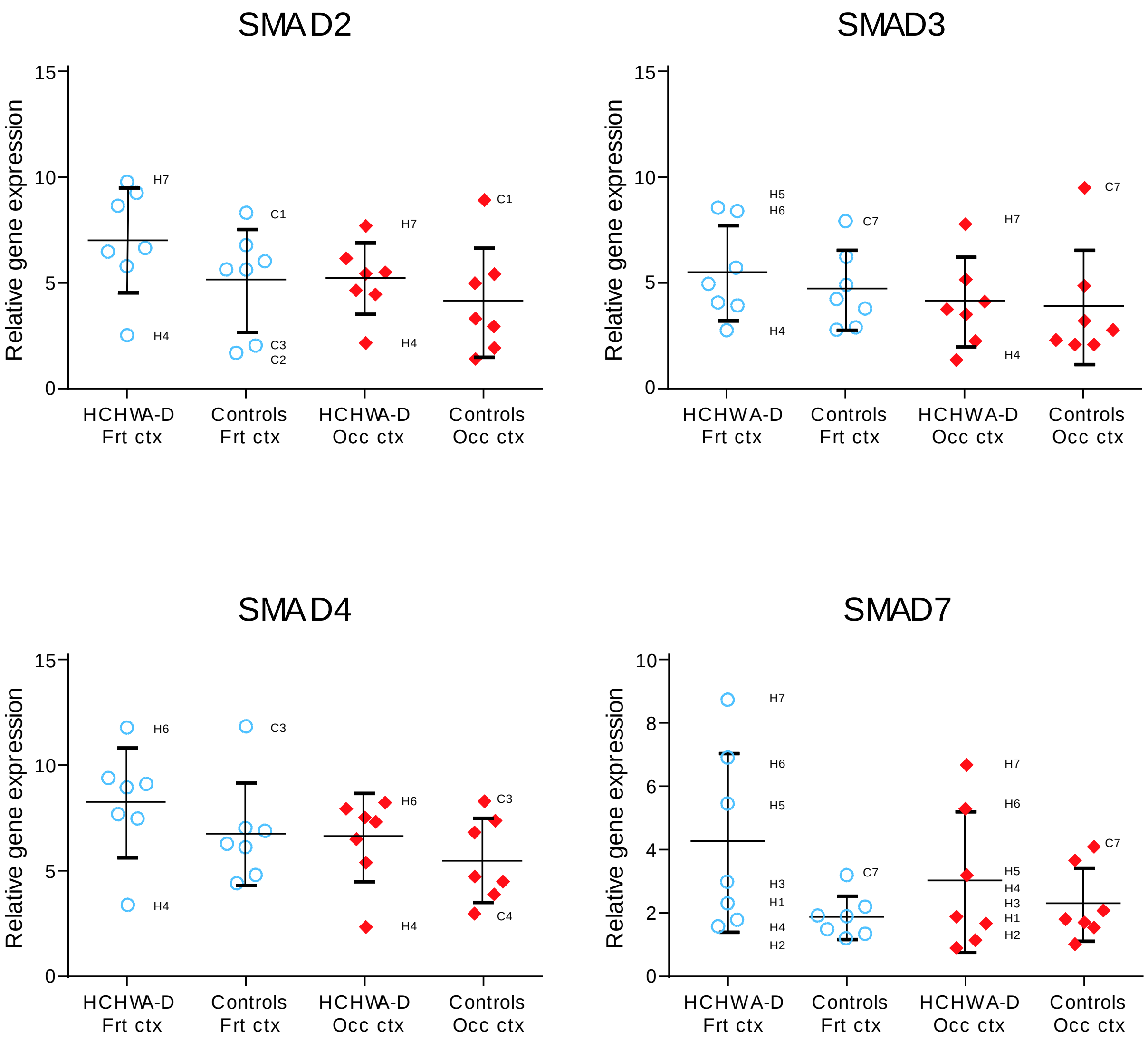

Supplementary Fig. 2 No significant upregulation of SMAD transcription factors gene levels in HCHWA-D frontal and occipital cortex compared to age-related controls. Transcript expression levels in postmortem brain cortex were normalized with two reference genes and represented in a dot plot with mean ± SD of 7 samples

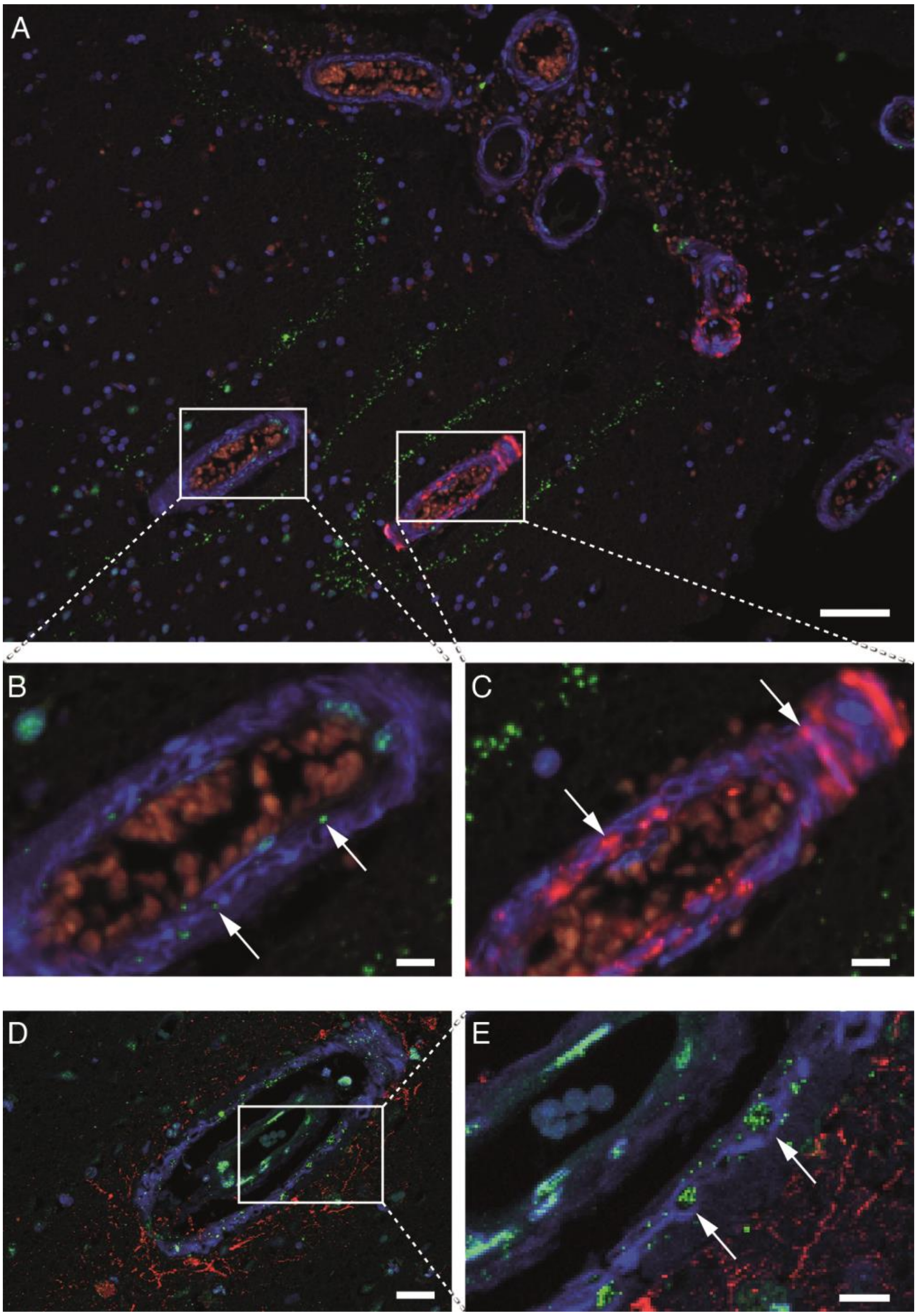

Supplementary Fig. 3 pSMAD2/3 granular deposits in angiopathic vessel walls are posterior to vascular smooth muscle cells (VSMCs) disappearance.

(**A**) Immunohistochemical pSMAD2/3 staining (pSMAD2/3 TSA (*green*), SMA (*red*) and nuclei (*blue*). (**B and C**) detail of (**A**). pSMAD2/3 granules are present on the vessel wall in the absence of smooth muscle actin (SMA) staining (*arrows*) (**B**); but were not colocalizing with SMA staining in remnant vascular smooth muscle cells (VSMCs) (*arrows*) (**C**). HCHWA-D H1 patient occipital cortex, epifluorescence microscope, Leica DM5500. *Scale bar* (**A**) 50nm (**B**;**C**) 10nm

(**D**) Immunohistofluorescent double staining with pSMAD2/3 (*green*), GFAP (*red*) and nuclei (*blue*). (**E**) detail of (**D**). pSMAD2/3 granular deposits are accumulating in the tunica media, here in vacuoles, believed to be the remains of the VSMCs (*arrows*) (**E**). HCHWA-D H2 patient-occipital cortex, merged confocal stack. *Scale bar* (**D**) 25nm (**E**) 10nm

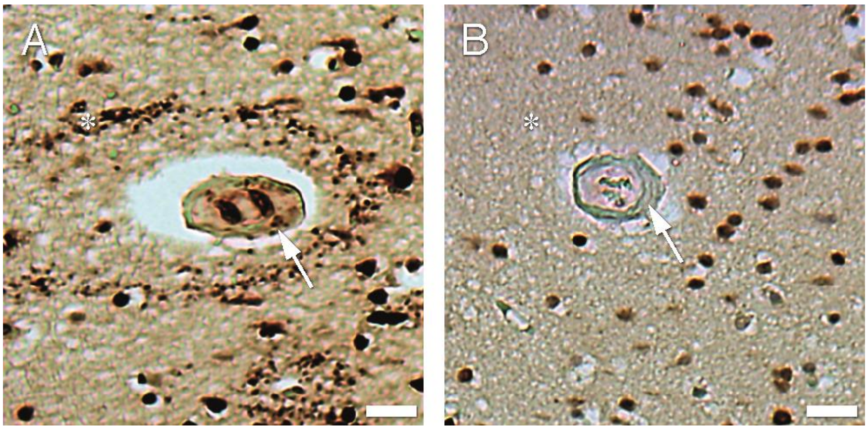

Supplementary Fig. 4 SMAD4 did not colocalize with pSMAD2/3 granules neither at the perivascular ring (*star*), nor on the vessel wall (*arrow*). (A) immunohistochemical pSMAD2/3 staining (TSA enhancement with DAB procedure, *brown*). (B) immunohistochemical SMAD4 staining (*brown)*. HCHWA-D H1 occipital cortex. *Scale bar* 25µm

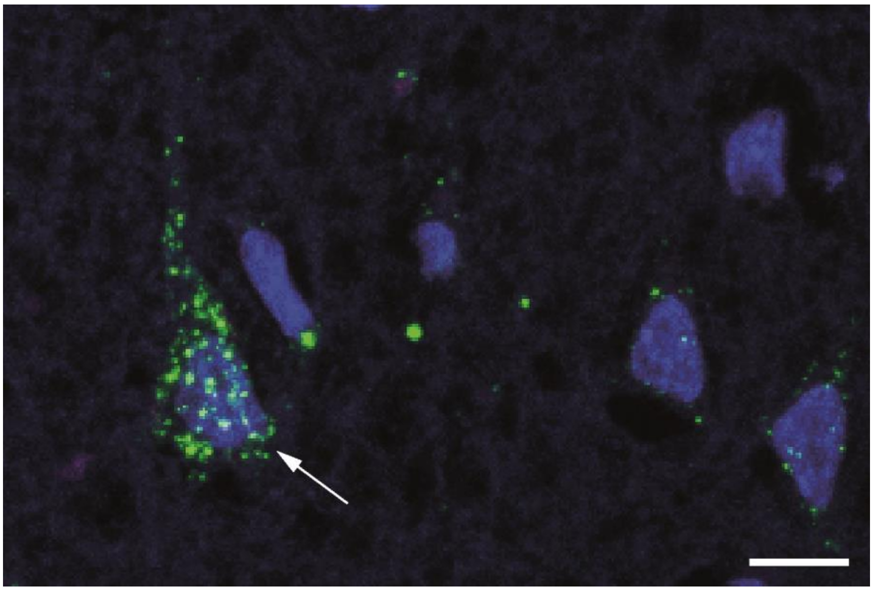

Supplementary Fig. 5 In HCHWA-D, a rare example of parenchymal cytoplasmic granulo-vesicular pSMAD2/3 (*arrow*) in a neuron (shape-based identification) associated with reduced nuclear signalling, as described in AD [2,6,38]. Immunohistofluorescent staining with pSMAD2/3 TSA (*green*) and nuclei (*blue*) channels. HCHWA-D H4 patient occipital cortex. *Scale bar* 10µm

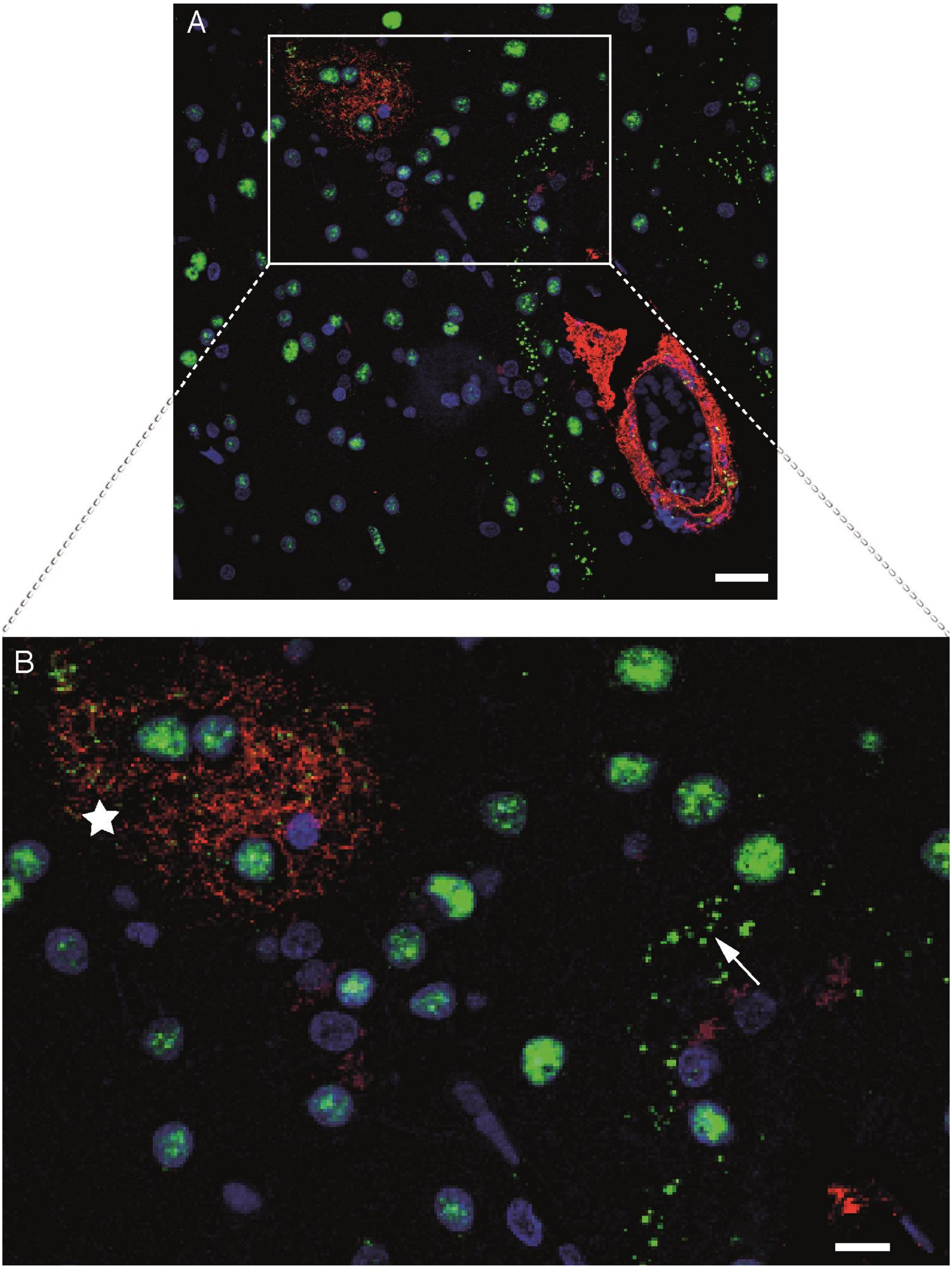

Supplementary Fig 6 Extra-cellular pSMAD2/3 deposits co-localized with diffuse parenchymal Aβ plaque (*star*), like in AD [6,38], but are different from the bright round-shaped dots composing the perivascular granular ring (*arrow*). Immunohistofluorescent double staining with pSMAD2/3 TSA (*green*), Aβ 6E10 (*red*) and nuclei (*blue*). (A) Merged confocal stack, (B) detail of (A). HCHWA-D H1 patient occipital cortex. *Scale bar (*A) 25μm; (B) 10μm
