# Supplementary material for: TGFβ pathway deregulation and abnormal phospho‐SMAD2/3 staining in hereditary cerebral hemorrhage with amyloidosis‐Dutch type
Source: Brain Pathol. 2017 Jun 12;28(4):495–506. doi: 10.1111/bpa.12533 (PMC8028662; doi:10.1111/bpa.12533)
Supplement: Supplementary file 2 — Table S1. List of antibodies used in this study. Table S2. Occurrence of pSMAD2/3 deposits in occipital cortex of HCHWA‐D subjects and clinical details. [file BPA-28-495-s001.pdf]

Supplementary tables

Article title: TGFβ pathway deregulation and abnormal phospho-SMAD2/3 staining in hereditary cerebral hemorrhage with amyloidosis-Dutch type

Journal: Brain Pathology (2017)

Authors and affiliations: Laure Grand Moursel*[1,2], Leon P. Munting[1,2], Linda M. van der Graaf[1,2], Sjoerd G. van Duinen[3], Marie-Jose T.H. Goumans[4], Uwe Ueberham[5], Remco Natté[3], Mark A. van Buchem[2], Willeke M.C. van Roon-Mom[1], Louise van der Weerd[1,2]

[1]Department of Human Genetics, Leiden University Medical Center
[2]Department of Radiology, Leiden University Medical Center
[3]Department of Pathology, Leiden University Medical Center
[4]Department of Molecular Cell Biology, Leiden University Medical Center
[5]Paul Flechsig Institute of Brain Research, University of Leipzig

****Corresponding author**
Leiden University Medical Center, Einthovenweg 20, 2333 ZC Leiden, The Netherlands
Phone: +31-71-526 9603 E-mail: L.Grand_Moursel@lumc.nl

Supplementary Table 1 list of antibodies used in this study

Reference	Name or clone	Antigen /Epitopes recognized	Host	Dilution IHC	Company & Type
3101S[a]	pSMAD2/3	Phospho-SMAD2 (Ser465/467)/SMAD3 (Ser423/425)	Rb (pc)	1:500 or 1:1000 +TSA	Cell Signaling
3108[a]	pSMAD2/3	Phospho-SMAD2 (Ser465/467)/SMAD3 (Ser423/425)	Rb (mc)	1:100	Cell Signaling
sc-11769-R[a]	pSMAD2/3	Phospho-SMAD2 (Ser465/467)/SMAD3 (Ser423/425)	Rb (pc)	1:500 or 1:1000 +TSA	Santa Cruz
9515	SMAD4	SMAD4	Rb (pc)	1:100	Cell Signaling
A1474	6E10	Aβ protein [1-17]	Ms (mc)	1:1000	Sigma
A1349	4G8[b]	Aβ protein [17-24]	Ms (mc)	1:1000	Sigma
G3893	GA5	Glial Fibrillary Acidic Protein (GFAP)	Ms (mc)	1:5000	Sigma
MAB364	HM-2	Microtubule-associated protein 2 (MAP-2)	Ms (mc)	1:2000	Chemicon
BR-003	AT-8	Human PHF-tau, phosphorylated at Ser202 and Thr205	Ms (mc)	1:2000	Innogenetics
mab1510	Ubi-1	Ubiquitin	Ms (mc)	1:3000	Millipore
ab6586	Collagen IV	Collagen IV native protein	Rb (pc)	1:200[c]	Abcam
ab11575	Laminin	Laminin native protein	Rb (pc)	1:100[d]	Abcam
M0851	SMA (IA4)	Smooth muscle actin	Ms (mc)	1:2000	Dako

Rb Rabbit, Ms Mouse, pc polyclonal, mc monoclonal

[a] the present study was conducted with #3101S antibody; sc-11769-R recognizing the same epitope gave exact identical pattern (Supplementary Figure 1C). #3108, a monoclonal version of #3101S, also stained the pSMAD2/3 granules although the signal was weaker (Supplementary Figure 1D).

[b] data not shown, identical to 6E10 staining

[c] no antigen retrieval

[d] proteinase K antigen retrieval

Supplementary Table 2 Occurrence of pSMAD2/3 deposits in occipital cortex of HCHWA-D subjects and clinical details

Code	Age	Gender	pSMAD2/3 angiopathic vessels[a]	Perivascular pSMAD2/3 granules[b]	Delay last ICH to death	Interval 1st stroke-death	Previous stroke	Cause of death
H7	71	M	77 % *	+/-	No major bleeding	26 y	No major bleeding	hematemesis
H6	61	M	70 % *	+/-	8 y	15 y	5	pneumonia
H4	55	F	72 %	+	3 d (operated)	3 d	0	ICH
H5	50	M	46 %	+	n.a.	6 y	2	ICH
H1	48	M	36 %	++	4 d	3 y	1	ICH
H2	57	M	3 %	-	17 d (remission)	9 y	2	pneumonia
H3	53	F	0 %	+/-	1 d	1 d	0	ICH
H8	51	M	43 %	+++	3 d	4 y	1	ICH
H11	81	F	39 %	- [#]	No major bleeding	23 y	No major bleeding	pneumonia
H9	67	F	84 % *	- [#]	unknown	21 y	3 (1 ischemic)	suffocation
H10	71	M	63 %	- [#]	1 d	17 y	3	ICH

ICH intracerebral hemorrhage, y years, d day(s), n.a. not available

[a] percentage from average grading occipital cortex, * capCAA involved

[b] ranked based on single slide observation (+/- : scarcely found, + to +++ : occurrence of finding), [#] DAB procedure only (although visible with DAB staining; the presence of perivascular granules is more easily assessed in immunofluorescence and preferably with TSA enhancement)
